# Different Current Intensities of Anodal Transcranial Direct Current Stimulation Do Not Differentially Modulate Motor Cortex Plasticity

**DOI:** 10.1155/2013/603502

**Published:** 2013-03-18

**Authors:** Dawson J. Kidgell, Robin M. Daly, Kayleigh Young, Jarrod Lum, Gregory Tooley, Shapour Jaberzadeh, Maryam Zoghi, Alan J. Pearce

**Affiliations:** ^1^Centre for Physical Activity and Nutrition Research, School of Exercise and Nutrition Sciences, Deakin University, Melbourne, VIC 3125, Australia; ^2^Cognitive and Exercise Neuroscience Unit, School of Psychology, Deakin University, Melbourne, VIC 3125, Australia; ^3^School of Physiotherapy, Monash University, Melbourne, VIC 3199, Australia

## Abstract

Transcranial direct current stimulation (tDCS) is a noninvasive technique that modulates the excitability of neurons within the motor cortex (M1). Although the aftereffects of anodal tDCS on modulating cortical excitability have been described, there is limited data describing the outcomes of different tDCS intensities on intracortical circuits. To further elucidate the mechanisms underlying the aftereffects of M1 excitability following anodal tDCS, we used transcranial magnetic stimulation (TMS) to examine the effect of different intensities on cortical excitability and short-interval intracortical inhibition (SICI). Using a randomized, counterbalanced, crossover design, with a one-week wash-out period, 14 participants (6 females and 8 males, 22–45 years) were exposed to 10 minutes of anodal tDCS at 0.8, 1.0, and 1.2 mA. TMS was used to measure M1 excitability and SICI of the contralateral wrist extensor muscle at baseline, immediately after and 15 and 30 minutes following cessation of anodal tDCS. Cortical excitability increased, whilst SICI was reduced at all time points following anodal tDCS. Interestingly, there were no differences between the three intensities of anodal tDCS on modulating cortical excitability or SICI. These results suggest that the aftereffect of anodal tDCS on facilitating cortical excitability is due to the modulation of synaptic mechanisms associated with long-term potentiation and is not influenced by different tDCS intensities.

## 1. Introduction

The excitability of cortical neurons within the primary motor cortex (M1) can be readily modified by the application of weak transcranial direct currents ranging from 0.2 mA up to 2 mA [1–4].

Transcranial direct current stimulation (tDCS) of the M1 elicits changes in cortical excitability in a polarity-specific manner. In general, anodal tDCS of long duration (i.e., up to 13 minutes) induces facilitatory effects of motor-evoked potentials (MEPs) [2, 3, 5], whilst cathodal tDCS leads to inhibitory effects [1, 6–9]. Specifically, single session tDCS with current intensities of at least 0.6 mA up to 2 mA applied for 5–20 minutes has been shown to modulate cortical excitability for up to 60 minutes after stimulation (see Bastani and Jaberzadeh [9]) and to have beneficial effects in a number of neurological conditions including stroke and Parkinson's disease [10–12].

The physiological mechanisms underlying anodal tDCS-induced cortical plasticity are not completely clear and may involve different mechanisms [3]. During anodal tDCS, the polarity of the resting membrane potential is altered, whereas any sustainable changes following the period of stimulation are likely to be mediated by physiological changes in synaptic activity [3, 13–15]. The mechanisms modulating the aftereffects of anodal tDCS on cortical excitability reflect changes in synaptic modification, sharing mechanisms similar to long-term potentiation, in particular the adjustment of *γ*-aminobutyric acid (GABAergic) and glutamatergic synapses [3, 13]. In support of this, a number of pharmacological investigations in humans have shown that the aftereffects of anodal tDCS are affected by drugs that alter neuronal membrane excitability or the efficacy of the N-methyl-D-aspartate (NMDA) receptor [13, 14, 16, 17]. Given this evidence it can be implied that the aftereffects of anodal tDCS, to some extent, share similarities with the mechanisms associated with use-dependant cortical plasticity [18, 19].

Although there is considerable evidence to show that anodal tDCS can increase cortical excitability [1, 2, 20], there is very little data describing the type of neurons that are modulated by different current intensities delivered by anodal tDCS. Early intracellular investigations showed that different direct current (DC) intensities target different cortical neurons, with weak stimulation potentially modulating predominantly nonpyramidal neurons, whilst stronger intensities presumably modulate pyramidal neurons [21]. Moreover, few studies have examined the effect of anodal tDCS on short-interval intracortical inhibition (SICI) and the effect of different DC intensities on modulating GABAergic inhibitory circuits with the M1. Given the clinical potential for tDCS [10–12], understanding the effects of different DC intensities on modulating GABAergic inhibitory systems is important, as GABA plays a critical role in shaping cortical excitability by minimising inappropriate activation of muscles that are not required for a movement task [22–24].

GABAergic inhibition in the M1 can be assessed noninvasively with paired-pulse transcranial magnetic stimulation or TMS [25]. When a subthreshold conditioning stimulus precedes a suprathreshold test stimulus by an inter-stimulus interval (ISI) of less than 5 ms, it results in suppressed MEPs compared to those from single-pulse stimuli at the same intensity [26]. The conditioning stimulus activates low-threshold inhibitory circuits that use the neurotransmitter GABA, resulting in synaptic inhibition of corticospinal neurons targeted by the suprathreshold test stimulus [27, 28]. The ratio between the amplitudes of the paired-pulse and single-pulse MEP responses represents SICI. Two distinct phases of inhibition have been described: one that occurs with an ISI of 1 ms and the other with an ISI of 2–4 ms. Little is known about the inhibition at 1 ms; however, it is now accepted that the inhibition occurring at an ISI of 2–4 ms is synaptic in origin, mediated by GABAergic inhibitory neurons acting via GABAA receptors [25, 29, 30].

With regards to the effects of anodal tDCS, it remains unclear what effect different DC intensities have on modulating SICI, with only a few studies investigating the effects of anodal tDCS applied at 1 mA [3, 10, 31]. In these studies, SICI was reduced immediately following 10–13 minutes of anodal tDCS [3, 10]. However, the effect of increased or decreased current intensity on modulating GABAergic interneurons is currently unknown. Therefore, the main aim of this study was to explore the effects of different common current intensities (0.8, 1.0 and 1.2 mA) and their aftereffects following anodal tDCS on cortical excitability and SICI. We recorded corticospinal activity evoked by single-pulse and paired-pulse TMS, with the primary aim to quantify the physiological effects that different anodal tDCS current intensities have on modulating cortical excitability and SICI and to describe the aftereffects of anodal tDCS on cortical excitability and SICI. We hypothesized that weak current intensities would increase intracortical inhibition, whilst stronger intensities would increase cortical excitability and the aftereffects would be larger due to less intracortical inhibition.

## 2. Materials and Methods

### 2.1. Participants

Fourteen healthy adults (range 22–45 years) without a history of upper limb injury or neurological disorder participated in the study. All participants were consistent right hand dominant (mean laterality quotient, 77.5 ± 18.5) according to the 10-item version of the Edinburgh Handedness Inventory [32]. Prior to the experiment, all participants completed the Adult Safety Screening Questionnaire to determine their suitability for TMS and tDCS application [33]. All participants gave written informed consent prior to participation in the study, which was approved by the Deakin University Human Research Ethics Committee. All experiments were conducted according to the standards established by the Declaration of Helsinki.

### 2.2. Experimental Design

Using a randomized, cross-over design, each participant underwent 10 minutes of three different conditions of constant anodal tDCS to the optimal cortical representation of left M1 for the right extensor carpi radialis longus (ECRL) muscle. The stimulation conditions were anodal tDCS delivered at either 0.8 mA, 1.0 mA, or 1.2 mA. The order of these conditions was counterbalanced and randomized across participants, with a wash-out period of one week between conditions. All participants and the lead researcher were blinded to the stimulation mode of tDCS across the three conditions. Single and paired pulse TMS was used to assess the aftereffects of anodal tDCS on the corticospinal excitability of the left M1. Ten single-pulse (120% of active motor threshold (AMT)) and 10 paired-pulse (70% of AMT) TMS stimuli were applied over the cortical area for right ECRL at baseline, immediately following, and 15 and 30 minutes following anodal tDCS, with the order of TMS stimuli (single or paired pulse) prior to and following tDCS, randomized throughout the trials (20 trials in total for each time point).

### 2.3. Transcranial Direct Current Stimulation of Primary Motor Cortex

Anodal tDCS was delivered by a battery-driven constant-current stimulator (neuroConn, Ilmenau, Germany) via a pair of conductive rubber electrodes, each positioned inside a saline-soaked surface sponge electrode (25 cm^2^). The stimulating electrode (anode) was fixed with two straps over the optimal cortical representation of the right ECRL muscle as identified by TMS over the left cortex, and the reference electrode (25 cm^2^) was placed over the right contralateral supraorbital area. The current was ramped up or down over the first and last five seconds, to avoid alternating currents causing transient neuronal firing [34]. In order to obtain the participants perception of discomfort throughout the tDCS current intensities, discomfort was assessed using a visual analog scale (VAS), with “no discomfort” at one end of a 100 mm line and “extremely uncomfortable” at the other, during the first three minutes of anodal tDCS.

### 2.4. Transcranial Magnetic Stimulation and Electromyography

Focal TMS was used to measure cortical excitability and SICI of the contralateral ECRL. Specifically, TMS was applied over the left M1 using a BiStim unit attached to two Magstim 200^2^ stimulators (Magstim Co, Dyfed, UK) to produce MEPs in the right ECRL. A figure-eight coil, with an external loop diameter of 9 cm, was held over the left M1 at the optimum scalp position to elicit MEPs in the right ECRL. The induced current in the brain flowed in a posterior-to-anterior direction. Sites near the estimated centre of the ECRL were explored to find the optimal site at which the largest MEP amplitude was obtained, and this area was marked by a small “x” in permanent marker. To ensure consistency throughout the study period and reliability of coil placement, the participants and researcher maintained the mark between experimental conditions. Care was taken by the researcher to ensure that the coil was held over the same position on the scalp so that the same area of the M1 was stimulated for all experimental conditions. All TMS measures were taken during weak voluntary contraction, by having the participant hold their hand in line with their wrist (neutral position). Root mean square (rms) electromyography (EMG) of the ECRL was obtained prior to each TMS stimulus to ensure that there were no changes in prestimulus rmsEMG which may have altered the MEP amplitude. Active motor threshold (AMT) was determined as the minimum stimulus intensity that produced a small MEP (200 *μ*V in 5 out of 10 consecutive trials) during isometric contraction of the ECRL at 5%  ± 2% of maximal rmsEMG activity [35]. A constant level of contraction was maintained with reference to an oscilloscope (HAMEG, Mainhausen, Germany) that displayed the rmsEMG signal in front of the participant. The stimulus intensity started at 50% of maximum stimulator output (MSO) and was altered in increments of ±1% of MSO until the appropriate threshold level was achieved. All MEP amplitudes were evaluated using an in test-stimulus intensity of 120% AMT.

Surface electromyography (sEMG) activity was recorded from the right ECRL muscle using bipolar Ag-AgCl electrodes. These electrodes were placed on the ECRL muscle, with an interelectrode distance (centre to centre) of 2 cm with a muscle belly-tendon montage. A grounding strap placed around the wrist was used as a common reference for all electrodes. All cables were fastened with tape to prevent movement artefact. The area of electrode placement was shaven to remove fine hair, rubbed with an abrasive skin rasp to remove dead skin, and then cleaned with 70% isopropyl alcohol. The exact sites were marked with a permanent marker by tracing around the electrode, and this was maintained for the entire three-week period by both the researcher and participant to ensure consistency of electrode placement relative to the innervation zone. An impedance meter was used to ensure impedance did not exceed 10 kΩ prior to testing. sEMG signals were amplified (×1000) with bandpass filtering between 20 Hz and 1 kHz and digitized at 2 kHz for 500 ms, recorded, and analyzed using PowerLab 4/35 (ADInstruments, Australia).

### 2.5. Short-Interval Intracortical Inhibition (SICI)

The protocol for SICI included 10 unconditioned stimuli, with a test intensity set to produce MEPs of ~1 mV in the ECRL and 10 conditioned stimuli, with a conditioning stimulus intensity set at 70% of AMT to induce SICI. Both AMT and the test stimulus intensity were adjusted at each time point following the removal of tDCS, if required, to ensure that AMT (% MSO) and the test MEP amplitudes were similar (1 mV) prior to and following tDCS. An ISI of 3 ms between the conditioning and test stimulus was used [25]. Single- and paired-pulse stimuli were presented according to a predetermined randomization protocol, with a 6–9 second time period between each stimulus.

### 2.6. Maximal Compound Muscle Action Potential

Direct muscle responses were obtained from the right ECRL muscle by supramaximal electrical stimulation (pulse width 1 ms) of the brachial plexus (Erb's point) under resting conditions (DS7A, Digitimer, UK). The site of stimulation that produced the largest M-wave was located by positioning the bipolar electrodes in the supraclavicular fossa. An increase in current strength was applied to the brachial plexus until there was no further increase observed in the amplitude of the sEMG response (*M*_MAX_). To ensure maximal responses, the current was increased an additional 20% and the average *M*_MAX_ was obtained from five stimuli, with a period of 6–9 seconds separating each stimulus.

### 2.7. Data Analyses

Prestimulus root mean square (rms) EMG activity was determined in the ECRL 100 ms prior to each TMS stimulus during each condition. Any prestimulus rmsEMG that exceeded 5%  ± 2% maximal rmsEMG was discarded and the trial repeated. The peak-to-peak amplitude of MEPs evoked as a result of stimulation was measured in the ECRL muscle contralateral to the cortex being stimulated in the period 10–50 ms after stimulation. MEP amplitudes were analysed using LabChart software (ADInstruments, Australia) after each stimulus was automatically flagged with a cursor, providing peak-to-peak values in *μ*V, and were then normalised to *M*_MAX_. Average MEP amplitudes were obtained for each trial for both single- and paired-pulse for each stimulation block (20 trials for each time point) separately. SICI was quantified by dividing the average paired-pulse MEP by the average single-pulse MEP (test intensity set to produce MEPs of 1 mV) and multiplying by 100.

Peak-to-peak amplitude and 30 ms (RMS) amplitude were automatically analysed from the M-wave separately for ECRL. The analysis was conducted by searching for the lowest and highest peaks in the EMG trace from a 30 ms epoch, which started 10 ms after the triggering of the percutaneous nerve stimulation. The lowest value was subtracted from the highest value to produce peak-to-peak M-wave value. Thereafter, the centre of the 30 ms RMS epoch was set to the mean between the highest and the lowest peak of the M-wave and RMS amplitude calculated from this epoch. In case that the beginning of the RMS epoch would have been less than 5 ms from the trigger, the beginning of the epoch was set at 5 ms from the trigger.

### 2.8. Statistical Analyses

Repeated measures ANOVA (independent variables: time course and current strength, dependent variables: MEP amplitudes and SICI) were used to calculate the effect of each tDCS condition (anodal tDCS at 0.8, 1.0, and 1.2 mA) on cortical plasticity. When appropriate, pairwise post hoc comparisons were carried out using Bonferroni correction to compare baseline cortical excitability and SICI prior to tDCS with those afterward. For all tests, the Huynh-Feldt correction was applied if the assumption of sphericity was violated. Alpha was set at *P* < 0.05, and all results are displayed as means ± SE.

## 3. Results

### 3.1. rmsEMG and VAS following Anodal tDCS

ANOVA was conducted on the average pretrigger rmsEMG calculated for each participant in the period 100 ms prior to single-pulse and paired-pulse TMS for each tDCS condition (0.8, 1.0, and 1.2 mA) and for the time points of baseline, immediately after, and 15 and 30 minutes following tDCS. Averaged over all conditions and time points, the mean pretrigger rmsEMG was 0.062 ± 0.016 mV. Pretrigger rmsEMG did not vary between single- and paired-pulse trails, or as a function of time, did not differ between conditions (*P* > 0.05) and all interactions were not significant (condition and time, all *P* > 0.05). Similarly, the perception of discomfort during tDCS did not vary between conditions (*P* > 0.05, Table 1).

### 3.2. Changes in Cortical Excitability with Anodal tDCS

**Figure 1:**
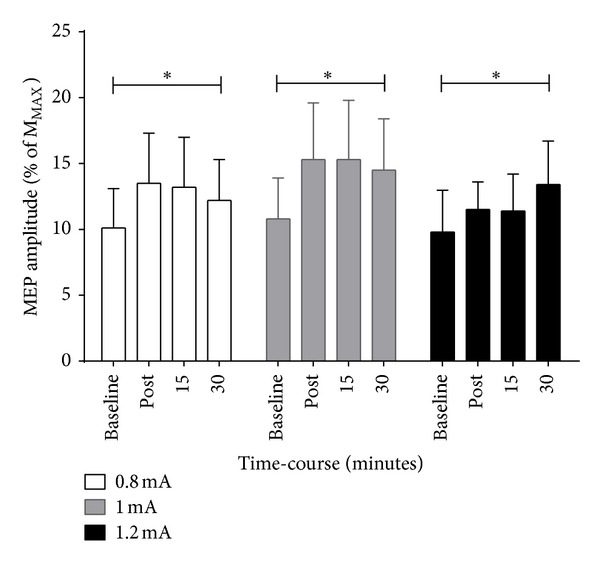
Mean ± SE motor-evoked potential amplitude (expressed as a percentage of *M*_MAX_) evoked in the ECRL muscle during baseline, immediately after, 15 and 30 minutes and for 0.8 mA (Left), 1.0 mA (middle) and 1.2 mA (right). Post hoc analyses showed that MEP amplitudes were facilitated immediately after and 15 and 30 minutes following anodal tDCS (*P* < 0.001); however there were no differences between current intensities (*P* > 0.05). *P < 0.05 compared to baseline.

Figure 1 presents MEP amplitudes expressed as a percentage of *M*_MAX_ measured in the ECRL for each condition (0.8 mA, 1.0 mA, and 1.2 mA) and by time (baseline, immediately after, and 15 and 30 minutes following that). ANOVA revealed that there was no significant condition-by-time interaction for cortical excitability (*F* = 2.98; *P* > 0.05). However, within each condition, there was a similar and significant time effect (*F* = 7.70; *P* < 0.001). Post hoc analysis showed a significant increase in cortical excitability immediately following and 15 and 30 minutes after anodal tDCS (all *P* < 0.001) compared to baseline for all conditions (see Table 2).

### 3.3. Changes in Short-Interval Intracortical Inhibition

**Figure 2:**
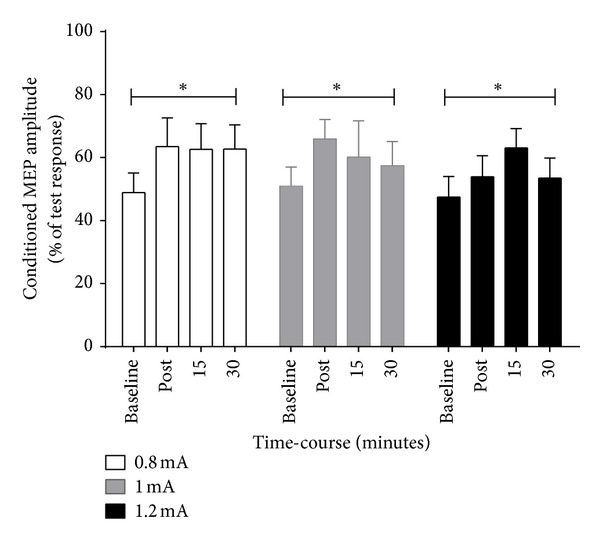
Mean ± SE short-interval intracortical inhibition (SICI) expressed as a ratio between conditioned MEPs and test MEPs measured in the ECRL muscle during baseline, immediately after, and 15 and 30 minutes following that for 0.8 mA (left), 1.0 mA (middle), and 1.2 mA (right). Post hoc analyses showed that SICI was reduced immediately post, 15 and 30 post tDCS (*P* < 0.001); however there were no differences between current intensities (*P* > 0.05). *P < 0.05 compared to baseline.

ANOVA revealed that there was no condition-by-time interaction (*F* = 1.30; *P* = 0.202), but over time SICI decreased (*F* = 7.83; *P* < 0.001, Figure 2). Post hoc comparisons showed significant reductions in SICI immediately following (48.9%  ±  6.2% to 68.8%  ±  9.4%; *P* < 0.001) and 15 (48.9%  ±  6.2% to 62.8%  ±  9.0%; *P* = 0.003) and 30 minutes (48.9%  ±  6.2% to 57.8%  ±  7.2%; *P* < 0.001) after anodal tDCS compared to baseline (Table 2).

## 4. Discussion

The purpose of this study was to examine the aftereffects of cortical excitability and intracortical inhibition, as an index of cortical plasticity, following different current intensities of anodal tDCS applied to M1. There were several important findings from this study. First, non-invasive cortical stimulation via anodal tDCS applied to the M1 increased cortical excitability and decreased intracortical inhibition that outlasted the stimulation period. Second, the magnitudes of change in MEP amplitude and intracortical inhibition were not different between conditions, illustrating that current intensities of 0.8 mA, 1.0 mA, and 1.2 mA with current densities of 0.032 mA/cm^2^, 0.040 mA/cm^2^, and 0.048 mA/cm^2^, respectively, do not differentially modulate cortical plasticity. Finally, because current density determines the efficacy of tDCS, we used a smaller electrode size (25 cm^2^) and a range of current densities to increase the focality of tDCS; however, this had no differential effects on cortical plasticity. Interestingly, there was also no difference in the perception of discomfort, which demonstrates that the participants were not aware of what current intensity was applied throughout the study period.

### 4.1. Change in MEP Amplitude after Anodal tDCS

An increase in MEP amplitude of the target muscle following anodal tDCS is thought to reflect cortical elements of plasticity [36, 37], via mechanisms associated with long-term potentiation in cortical circuits [18, 19, 36]. A similar increase in MEP amplitude has been observed following anodal tDCS in healthy participants, particularly when 1 mA is applied via 35 cm^2^ surface electrodes [1, 3, 13, 17]. A change in MEP amplitude of 40% (immediately after) that remains elevated for up to 60 minutes has been reported and confirmed by mathematical model that show tDCS can modify the transmembrane potential [38, 39], which influences the excitability of individual neurons.

Of particular interest to the current study was the effect of tDCS current intensity on modulating cortical plasticity. We hypothesised that different current intensities would differentially modulate cortical excitability, with low current intensities potentially increasing intracortical inhibition and higher current intensities having greater effects on MEP responses. Our findings that anodal tDCS current intensities of 0.8, 1.0, and 1.2 mA do not differentially modulate cortical excitability at any time period suggest that the same cortical circuits were stimulated to a similar magnitude during anodal tDCS of different current intensities and that the mechanisms involved in the aftereffects for all intensities were similar. Consistent with previous investigations, the results of the application of anodal tDCS of different intensities appear to evolve during stimulation, which most likely involves modification of the transmembrane potential [38], which is critical for inducing cortical plasticity via modulating NMDA-receptor efficacy [3, 13, 14].

Although there are limited reports within the literature regarding the aftereffects of different current intensities on cortical excitability, the increases in MEP amplitudes in the current study are comparable to the seminal study by Nitsche and Paulus [2], but with a number of important differences. The present study showed that cortical excitability remained facilitated 30 minutes following stimulation compared to only 4 minutes after tDCS shown by Nitsche and Paulus [2], and this is independent of current intensity (albeit a narrow range). Additionally, our findings demonstrate that the increase in cortical excitability is likely to be modulated by changes in intracortical inhibitory neurons. We observed a marked reduction in intracortical inhibition, which remained attenuated even at 30 minutes after tDCS. We speculate that the increase in MEP amplitude following tDCS could well be the consequence of a reduction in SICI because of the direct effect of tDCS on superficial intracortical inhibitory neurons [3, 10]. Certainly, there is good evidence for altered intracortical GABAergic inhibition stimulating changes in cortical excitability that have been found in other models of cortical plasticity [40–43].

### 4.2. Changes in SICI following Anodal tDCS

There have been limited investigations to describe the effects of tDCS on the GABAergic inhibitory systems [3, 10], and no studies have investigated the effect of different current intensities modulating intracortical inhibition. We have shown that SICI is reduced for up to 30 minutes following anodal tDCS and this decrease is independent of current intensity. Based upon this finding, we suggest that current intensities from 0.8 mA up to 1.2 mA primarily affect the excitability of intracortical inhibitory neurons, which subsequently increases cortical excitability. Importantly, modulation of intracortical inhibition, in this instance SICI, represents a candidate mechanism underlying the changes in cortical excitability [44, 45]. It is likely that tDCS removes local inhibition that impinges upon preexisting excitatory synapses onto corticospinal neurons; therefore producing any increase in MEP amplitude is due to the reduction in intracortical inhibition.

Understanding the effects of tDCS on intracortical inhibition is important as modulation of SICI is crucial for the selective activation of muscles, making it critical for accurate and coordinated motor function [23, 24]. Several studies suggest that paired-pulse TMS inhibition is dependent on the activity of inhibitory interneurons that act upon the postsynaptic GABAA receptors [46–48]. Certainly, several pharmacological interventions indicate that the inhibitory circuits can be assessed noninvasively by paired-pulse TMS. Our findings are consistent with previous studies [3, 10], whereby SICI is reduced following anodal tDCS. In contrast to our original hypothesis, there was no interaction between the type of current intensity and the modulation of GABAergic inhibition. This is consistent with the findings by Nitsche et al. [3], who reported that the aftereffects of SICI were predominantly modulated by anodal tDCS; however this was not affected by current intensity. The reduction in SICI can be interpreted as disinhibition of corticospinal neurons controlled by SICI circuits. This result is supported by previous literature [6, 49] that the aftereffects of tDCS are regulated by NMDA-receptor activity [14].

The current results also show that the amplitude of MEPs from the test response of the SICI inducing paired-pulse protocol was facilitated for up to 30 minutes following tDCS, independent of current intensity. Therefore, the ratio between the conditioned and unconditioned MEP responses increased following tDCS, indicating that there may be plasticity occurring as a result of tDCS reducing inhibition confined to the M1. Cortical projections to the ECRL are likely to be suppressed under normal conditions because of inhibitory mechanisms; however tDCS has altered the sensitivity of inhibitory cortical interneurons, thus reducing the efficacy of the connections between interneurons and corticospinal neurons [50, 51]. The potential mechanisms likely include the unmasking of silent synapses (disinhibition) confined to the M1 and synaptic plasticity at a cortical level [19, 36, 52, 53]. The reduction in SICI indicates a lesser amplitude of inhibitory postsynaptic potentials. Importantly, this allows corticospinal neuron membrane potentials to become closer in proximity to their firing thresholds, thus enhancing the excitatory synaptic strength during conditions of lowered inhibition. Taken together, the results of this study demonstrate a noticeable involvement of intracortical synaptic mechanisms that modulate cortical excitability. Our results further support a prominent role for tDCS modulating NMDA-receptor efficacy, as both intracortical inhibition and cortical excitability are controlled to some degree by this receptor. This finding is consistent with a number of previous investigations [13, 14], but Siebner et al. [31] showed no aftereffects of tDCS on intracortical inhibition.

Many plasticity inducing interventions, such as motor skill training, show that the removal of SICI is an important mechanism for optimal motor skill learning and in inducing motor cortex plasticity [40, 43, 54]. tDCS studies have been advocated to act as a potential primer for improving motor function [55]. In healthy adults, anodal tDCS has been shown to improve both gross and fine motor skill tasks [4, 56–59]. Further, in conditions such as stroke, anodal tDCS over the affected M1 has resulted in improvements in force production, pinch force, serial reaction, time and the Jebsen-Taylor Hand Function Test of the corresponding upper limb, which appear to correlate with increases in cortical excitability and decreases in SICI [10, 60–62]. Although we did not measure motor function, our findings of reduced intracortical inhibition and facilitation of cortical excitability are important as Hummel et al. [10] showed a significant relationship between the change in cortical plasticity and improvement in motor function (*r* ^2^ = 0.61). Interestingly, they reported a significant reduction in SICI following tDCS. Although the improvements in motor performance following tDCS are promising, the modification in cortical plasticity that usually occurs following training is not always associated with the level of behavioural improvement [63]. Further studies are required to determine the association between tDCS-generated plasticity and motor performance.

### 4.3. Limitations

Based upon the experimental design, there are some limitations that may have contributed to the lack of differential modulation of cortical excitability. For example, we did not collect input/output (IO) curves throughout our study, which provide important information about the spatial distribution of the excitable elements of the corticospinal tract. In particular, they serve as an index of the excitability of larger neuronal populations, as quantified by changes in the slope function. Recently, tDCS has been shown to increase the slope of IO curves [3, 10]. It is also likely that stimulating at only 120% AMT may not have been a sensitive enough measure to disassociate the effects of different current intensities in differentially adjusting cortical excitability [64]. Indeed, IO curves have proven sensitive in identifying changes in cortical excitability following different motor tasks when compared to simply using a stimulus intensity of 120% motor threshold [64].

The electrode size used may also have influenced the findings of the present study. For example, it is well known that the direct functional effects of tDCS are restricted to the area under the electrode and the strength of the electrical field is relatively uniform [38], with increasing electrode size reducing its focality. However, we observed a similar increase in MEP amplitude (33% immediately following) when using 25 cm^2^ electrodes to previous studies showing increased MEP amplitudes when tDCS is delivered via 35 cm^2^ electrodes [1, 3, 4]. There are at least two possibilities that could help explain why we have not observed larger MEP amplitudes with a more focal electrode size. First, it is possible that factors restricted to the participant population of the present study may have contributed to the similar magnitude of MEP change to previous studies, such as the extent of skilled hand use [65], prior history of synaptic activity [65], genetic factors (e.g., brain derived neurotrophic factor gene) [66], and gender, with the potential effect of the menstrual cycle [67]. Secondly, whilst we used a smaller stimulation electrode size, the size of the reference electrode was not modified, and evidence shows that reducing the size of the stimulating electrode and increasing the size of the reference electrode may further increase cortical excitability [68]. Another limitation is that the aftereffects of tDCS were only obtained up to 30 minutes following tDCS. Certainly, our data shows that even at 30 minutes, MEP responses (single- and paired-pulse MEPs) were still significantly facilitated compared to baseline. In this regard, we are unable to identify how long the aftereffects remain before returning to baseline. Finally, the narrow range of current intensities tested may explain why cortical excitability and inhibition were not differentially modulated. However, we specifically chose these ranges as they have been the most common current intensities used to modulate cortical excitability [9]; however there have been no reports on the effects of these intensities on SICI.

In conclusion, previous TMS studies have shown that tDCS modulates cortical plasticity in a polarity-dependent manner, but no studies have examined the effects of different current intensities on modulating the aftereffects of cortical excitability and intracortical inhibition. We found that the extent of cortical plasticity (the change in MEP amplitude and SICI) was facilitated for 30 minutes and this was independent of current intensity. The magnitude of change in MEP amplitude and intracortical inhibition was not different between conditions, illustrating that current intensities between 0.8 and 1.2 mA with current densities between 0.032 mA/cm^2^ and 0.048 mA/cm^2^ do not differentially modulate cortical excitability. These results show that current intensities between 0.8 mA and 1.2 mA primarily affect superficial intracortical inhibitory neurons, which increases cortical excitability, via the reductions in SICI. These findings have important clinical applications, by demonstrating that lower current intensities are just as effective in modulating cortical plasticity as higher intensities. However, further studies are needed to identify the effect of different tDCS current intensities on modulating use-dependent plasticity following motor skill training.

## Figures and Tables

**Table 1:** Mean ± SE descriptive data prior to anodal tDCS and mean ± SE visual analogue scale obtained during the first 3 mins of anodal tDCS during each current intensity.

Participant characteristics	Mean ± SE
Age (yrs)	27.5 ± 7.7
Weight (kg)	73.0 ± 14.6
Height (cm)	162.7 ± 42.3
Handedness (LQ)	77.4 ± 18.4
AMT (% MSO)	30.3 ± 6.5
Test intensity (% MSO)	35.7 ± 8.8
CS intensity (% MSO)	21.21 ± 5.3
M-wave (mV)	15.2 ± 3.1
VAS (mm) 0.8 mA	18.8 ± 6.0
VAS (mm) 1.0 mA	20.0 ± 7.0
VAS (mm) 1.2 mA	26.6 ± 5.1

**Table 2:** Mean ± SE cortical excitability and short-interval intracortical inhibition (SICI) responses following anodal tDCS: MEP amplitude at 120% AMT as a percentage (% of *M*_MAX_), and conditioned MEP amplitudes as a percentage of the test response (SICI ratio).

tDCS condition	Baseline	Post	15 min post	30 min post
MEP amplitude at 120% AMT (% of *M*_MAX_)				

0.8 mA	10.1 ± 3.0	13.5 ± 3.8	13.2 ± 3.8	12.2 ± 3.1
1.0 mA	10.8 ± 3.1	15.3 ± 4.3	15.3 ± 4.5	14.5 ± 3.9
1.2 mA	9.8 ± 3.17	11.5 ± 2.1	11.4 ± 2.8	13.4 ± 3.3

SICI (% of test response)				

0.8 mA	48.9 ± 6.2	63.5 ± 9.1	62.6 ± 8.2	62.7 ± 7.7
1.0 mA	50.9 ± 6.1	65.9 ± 6.2	60.1 ± 11.6	57.4 ± 7.7
1.2 mA	47.5 ± 6.5	53.9 ± 6.7	63.1 ± 6.1	53.5 ± 6.4
